# Supplementary material for: dFLASH; dual FLuorescent transcription factor activity sensor for histone integrated live-cell reporting and high-content screening
Source: Nat Commun. 2025 Apr 7;16:3298. doi: 10.1038/s41467-025-58488-w (PMC11977238; doi:10.1038/s41467-025-58488-w)
Supplement: Supplementary file 1 — Supplementary Information [file 41467_2025_58488_MOESM1_ESM.pdf]

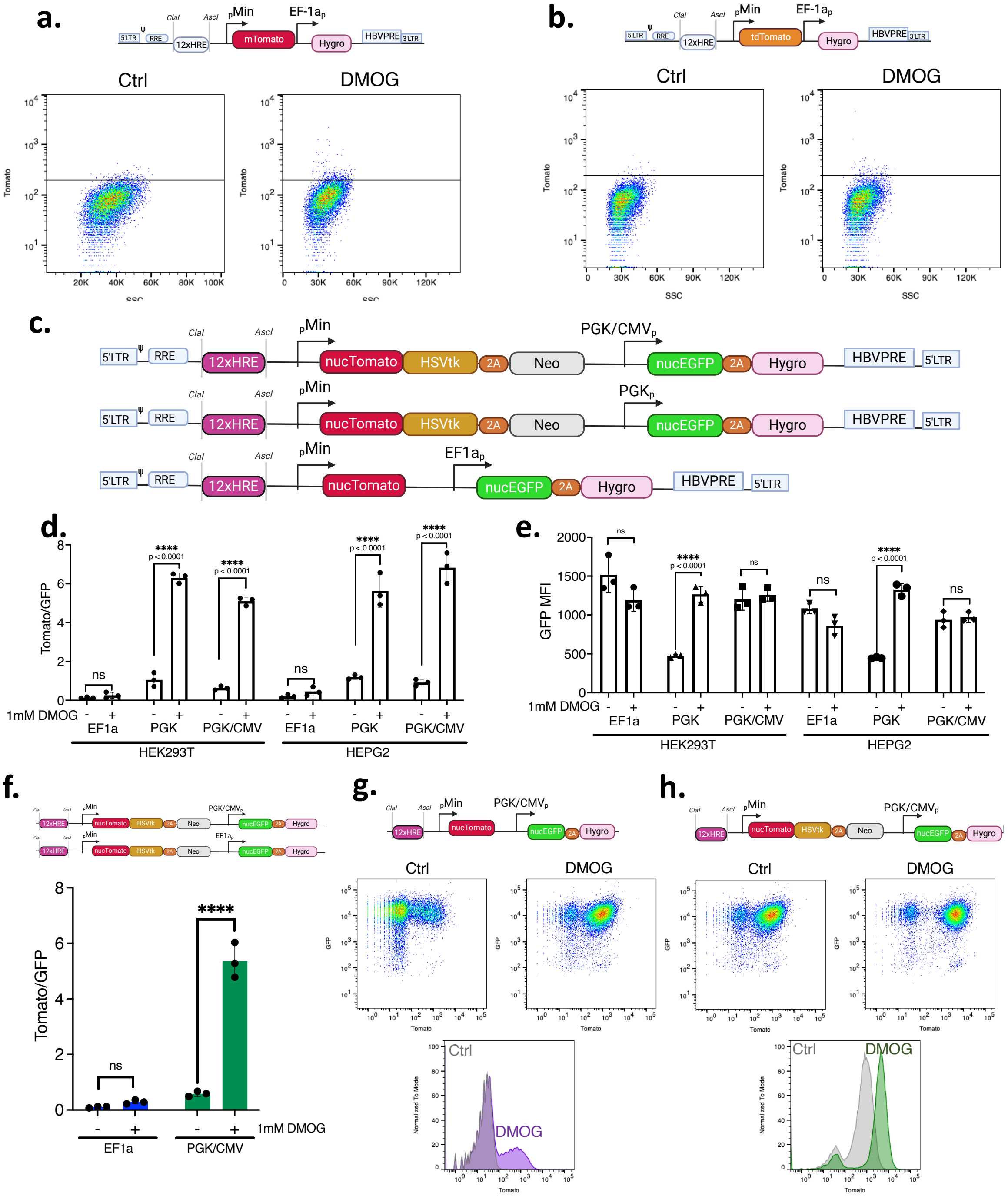

Supplementary Fig. 1. Optimised dFLASH design produces a robust HIF sensor.
(**a-b**) HEK293T cells with FLASH constructs without EGFP and (**a**) expressing monomeric Tomato or (**b**) dimeric Tomato were treated -/+ 1 mM DMOG for 48 hours and quantified by FACS. Tomato MFI >200 AU was used to compare induction (black line). (**a-b**) Created in BioRender. Peet, D. (2025) https://BioRender.com/f29a858 (**c-e**) HEK293T and HEPG2 cells were transduced with HRE-dFLASH reporters that had different downstream promoters controlling EGFP or Tomato cassette composition and treated for 48 hours -/+ 1 mM DMOG prior to HCI. (**c**) Created in BioRender. Peet, D. (2025) https://BioRender.com/f29a858 . (**d**) Tomato/EGFP MFI ratio and (**e**) EGFP MFI for each backbone variant was then compared (n = 3 biological replicates, presented as mean ± SD). (**f**) HEK293T cells transduced with reporter constructs containing the downstream PGK/CMV or EF1α promoters were compared for DMOG induction by HCI after 48 hours of -/+ 1 mM DMOG treatment (n = 3 biological replicates, presented as mean ± SD). Significance in **d**, **e**, and **f** assessed via Two-Way ANOVA (**** $p < 0.001$, ns = not significant). (**g,h**) HEK293T cells with the HRE enhancer and different dFLASH compositions (**g**) PGK/CMV dFLASH with Tomato alone as the upstream cassette or (**h**) with the tomato-HSVtK-Neo upstream cassette, treated for 48-hours -/+ 1 mM DMOG prior to analysis of EGFP and Tomato induction by FACS. (**f-h**) Created in BioRender. Peet, D. (2025) https://BioRender.com/f29a858 . Source data are provided as a Source Data file.

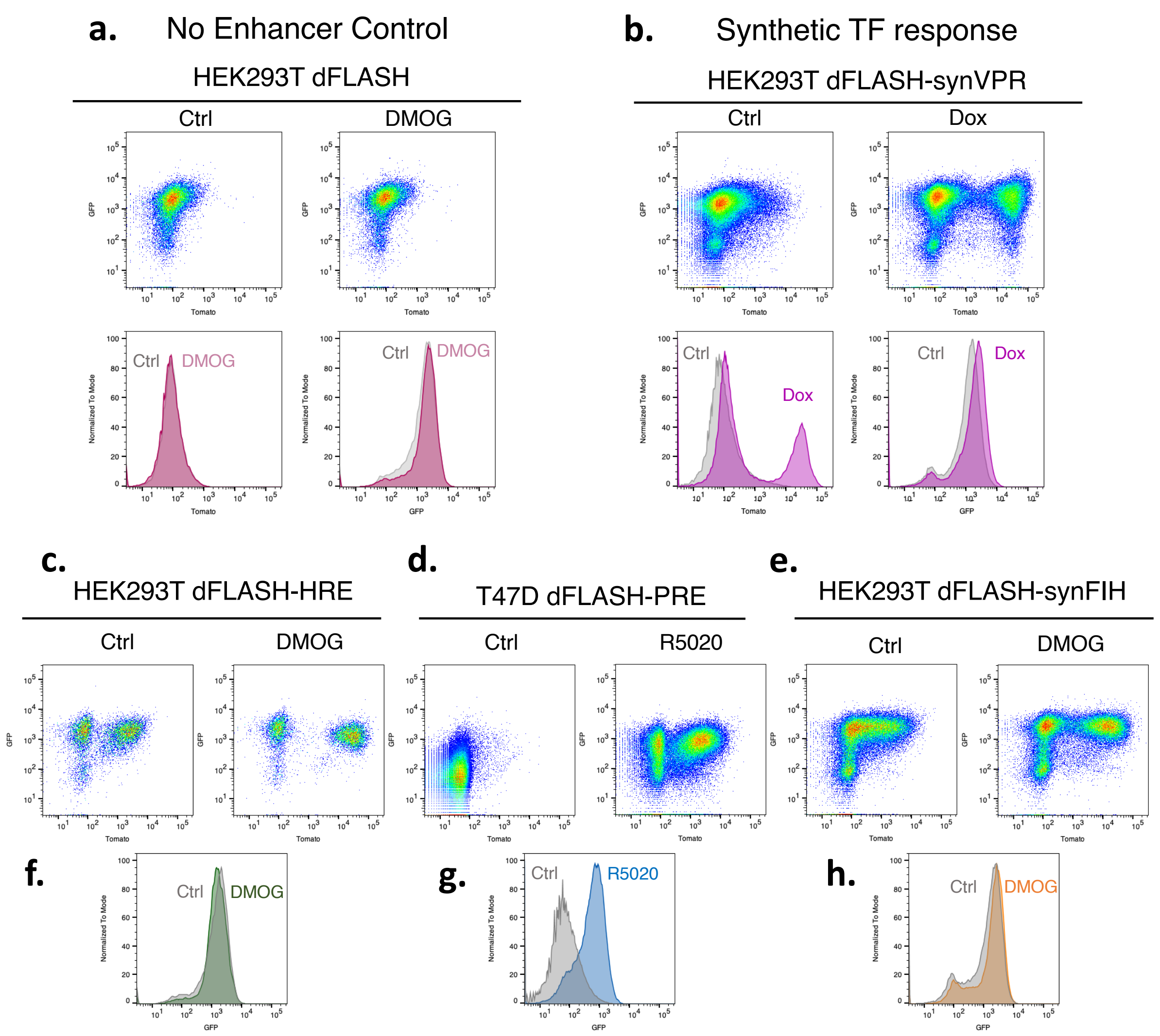

Supplementary Fig. 2. dFLASH provides a TF-responsive, versatile reporter platform in heterogenous cell pools.

(**a-b**) HEK293T cells were transduced with (**a**) dFLASH with no enhancer and treated with 1 mM DMOG or 0.1% DMSO (Ctrl) or (**b**) GalRE-dFLASH and Gal4DBD-miniVPR and treated with H_2O (Ctrl) or 1 μg/mL Dox for 48 hours prior to FACS. Dot plots of populations' Tomato and EGFP intensity with or without activating chemicals and histograms comparing EGFP and Tomato MFI between control and treated populations are shown. (**c-h**) Dot plots and EGFP histograms for control and chemical treated (**c, f**) dFLASH-HIF, (**d, g**) dFLASH-PR polyclonal pools (*to accompany **Fig. 2a-c***) and (**e, h**) dFLASH-synFIH.

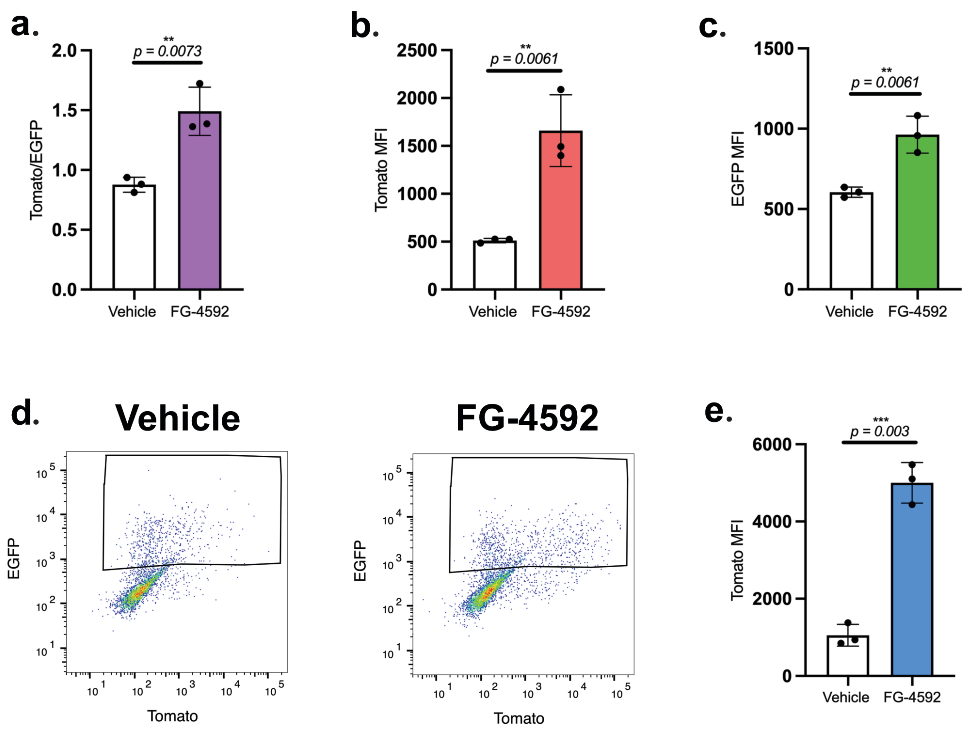

Supplementary Fig. 3. Application of dFLASH-HRE in primary mouse granulosa cells.
(**a-c**) High content imaging of dFLASH-HRE transduced mouse granulosa cells after 24 hours of 50 μM FG-4592 or vehicle (DMSO) stimulation. (**a**) Tomato normalized to EGFP, (**b**) Tomato Mean Fluorescent Intensity (MFI), (**c**) EGFP MFI. (**d**) Flow cytometry gating strategy to select EGFP positive dFLASH-HRE mouse granulosa cells. Representative plots from one of three biological replicates shown. (**e**) Tomato MFI of EGFP positive mouse granulosa cells, as gated in (**d**) after treatment with 50 μM FG-4592 or vehicle (DMSO) for 24 hours and analysed via flow cytometry. Data presented as mean ± standard deviation, n = 3 biological replicates. Significance assessed by t-test assuming equal standard deviation (** $p < 0.01$, *** $p < 0.0001$). Source data are provided as a Source Data file.

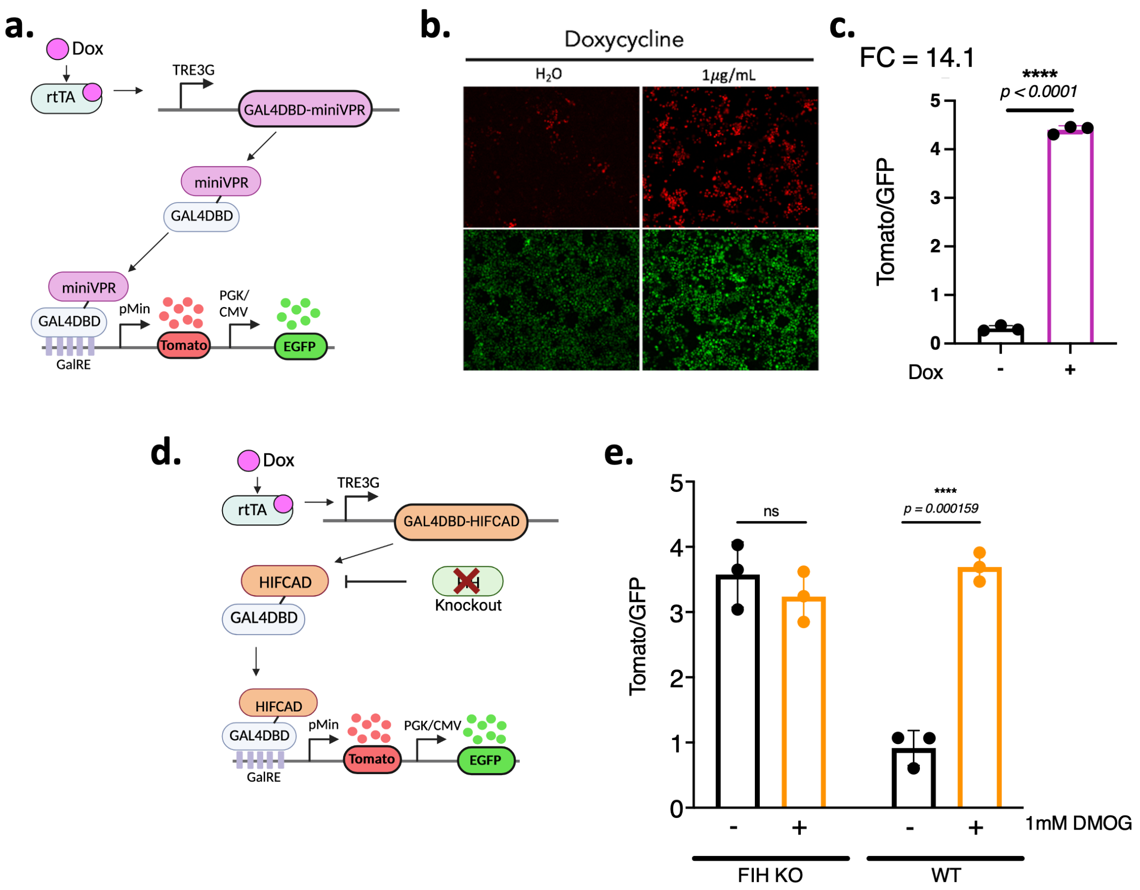

Supplementary Fig. 4. Synthetic transcription factors drive a strong response from the GalRE-dFLASH reporter and can respond to endogenous signaling pathways.

(**a**) GAL4DBD-miniVPR is expressed from an independent dox-inducible vector that subsequently binds to GRE-dFLASH (rtTA; reverse tetracycline controlled transactivator, GalRE; gal response element, gal4DBD; gal4 DNA binding domain). Created in BioRender. Peet, D. (2025) https://BioRender.com/o32v671 . (**b**,**c**) HEK293T GalRE-dFLASH cells were transduced with the doxycycline inducible GAL4DBD-miniVPR expression construct and treated -/+ doxycycline for 48 hours prior to HCI for (**b**) Tomato expression (top panels) and EGFP expression (bottom panels) and (**c**) quantification by HCI presented as normalised fluorescence intensity (n = 3 biological replicates, presented as mean ± SD). FC is fold change between the populations. (**d, e**) To confirm HEK293T dFLASH-synFIH system was FIH dependent, (**d**) GalRE-dFLASH and GAL4DBD-HIFCAD vectors were transduced into HEK293T cells with FIH knocked out. (**e**) FIH KO cells were compared with wildtype HEK293T dFLASH-synFIH (WT) in a 200 ng/mL Dox background for DMOG-dependent reporter induction by HCI (n = 3 biological replicates, presented as mean ± SD). (**c, e**) Significance was assessed by t-test with Welch's correction (ns = not significant, *** p <0.001, ****p <0.0001). Source data are provided as a Source Data file.

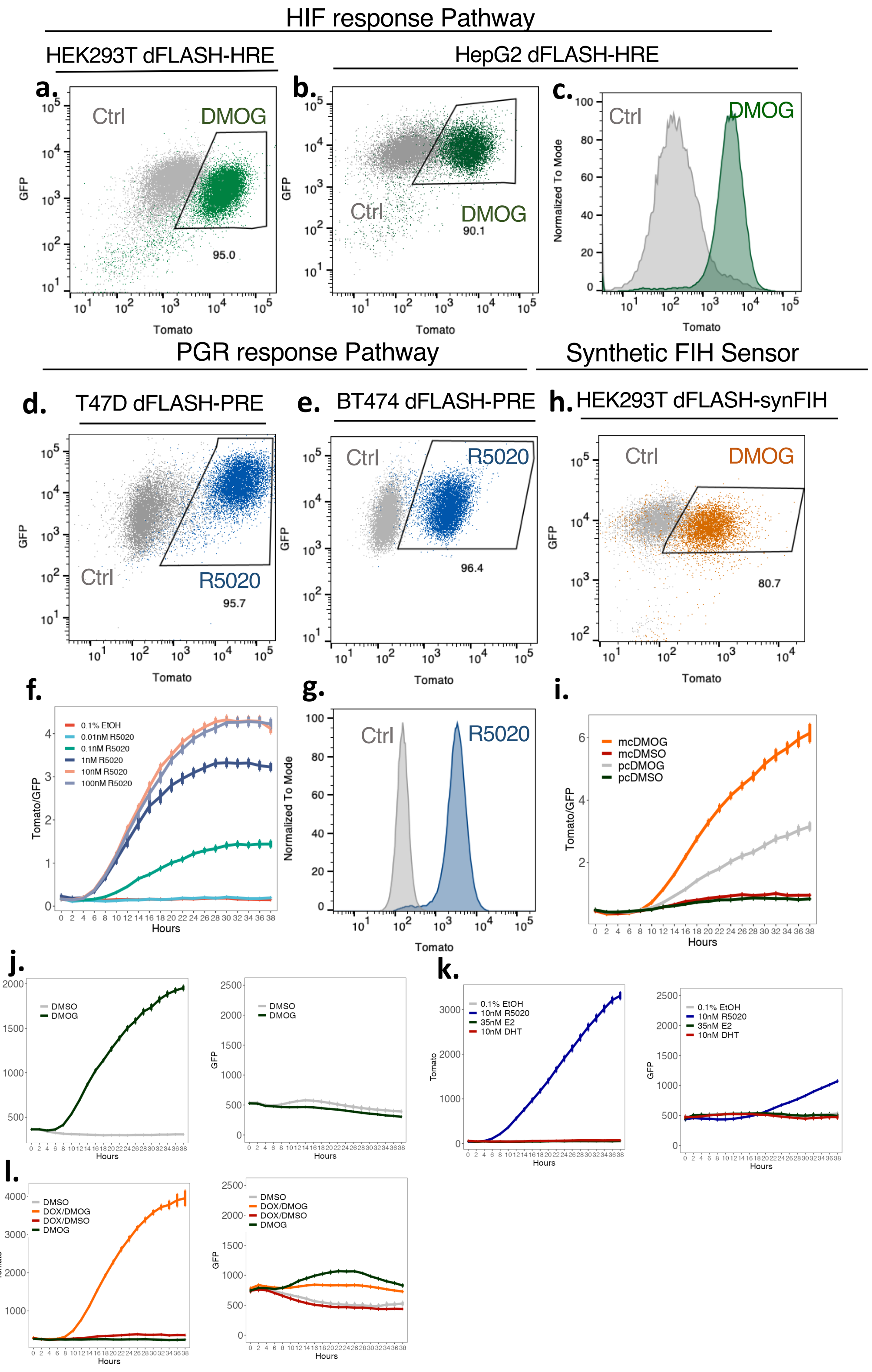

Supplementary Fig. 5. Clonal dFLASH cell lines enable improved reporting across different cell types.

(**a-c**) Flow cytometry of clonal dFLASH-HIF cell lines for (**a**) HEK293T (see also **Fig. 3b**) and (**b,c**) HepG2 cells after 48 hours -/+ 0.5 mM DMOG. (**d-g**) dFLASH-PGR functionality was assessed by flow cytometry in (**d**) T47D (see also **Fig. 3f**) and (**e,g**) BT474 cells after 48 hours -/+ 100 nM R5020. Percentage of Tomato-positive cells in ligand-treated populations are displayed. (**f**) T47D dFLASH-PGR cells were treated with increasing concentrations of R5020 (0.01-100nM, n=8 biological replicates per group) and imaged over 38 hours by temporal HCI to determine sensitivity to R5020. (**i**) Temporal HCI comparisons between monoclonal (mc) and polyclonal (pc) HEK293T dFLASH-synFIH cells (see also **Fig. 2j**). (**j-l**) Tomato and GFP high-content imaging time courses from Fig. 3 and Supplementary Fig. 5i for (**j**) HEK293T mcdFLASH-HRE, (**k**) T47D mcdFLASH-PRE and **(l)** HEK293T mcdFLASH-SynFIH. In (**f**) and (**i-l**) n = 4 biological replicates per group, data presented as mean ± sem. Source data are provided as a Source Data file.

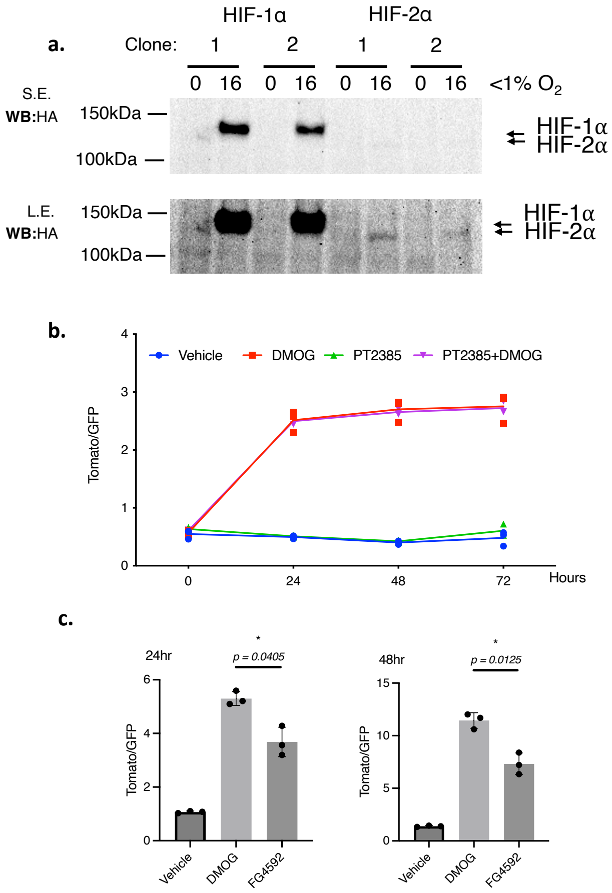

Supplementary Fig. 6. HIF-1α is the predominant isoform that controls the dFLASH reporter in HEK293T cells

(**a**) Monoclonal HEK293T cells with endogenously HA-Flag tagged HIF-1α or HIF-2α were treated with hypoxia (<1% O_2) for 16 hours prior to anti-HA immunoblotting of whole cell extracts. S.E.= short exposure; L.E.= long exposure. Representative of n =3 independent experiments. (**b**) mcdFLASH-HIF cells were treated -/+ 1 mM DMOG and -/+ 10 μM of the HIF-2α antagonist (PT-2385) as indicated and quantified by HCI over a 72-hour period, (n = 3 biological replicates). (**c**) mcdFLASH-HIF cells were treated with 50 μM FG-4592 or 1 mM DMOG and imaged by HTS-HCI at (left panel) 24 hours and (right panel) 48 hours. Mean ± sem of each group shown, data from n=3 independent experiments, four replicates per group. Significance was assessed with a Welch ANOVA and Dunnetts post-Hoc comparison (* $p < 0.05$). Source data are provided as a Source Data file.

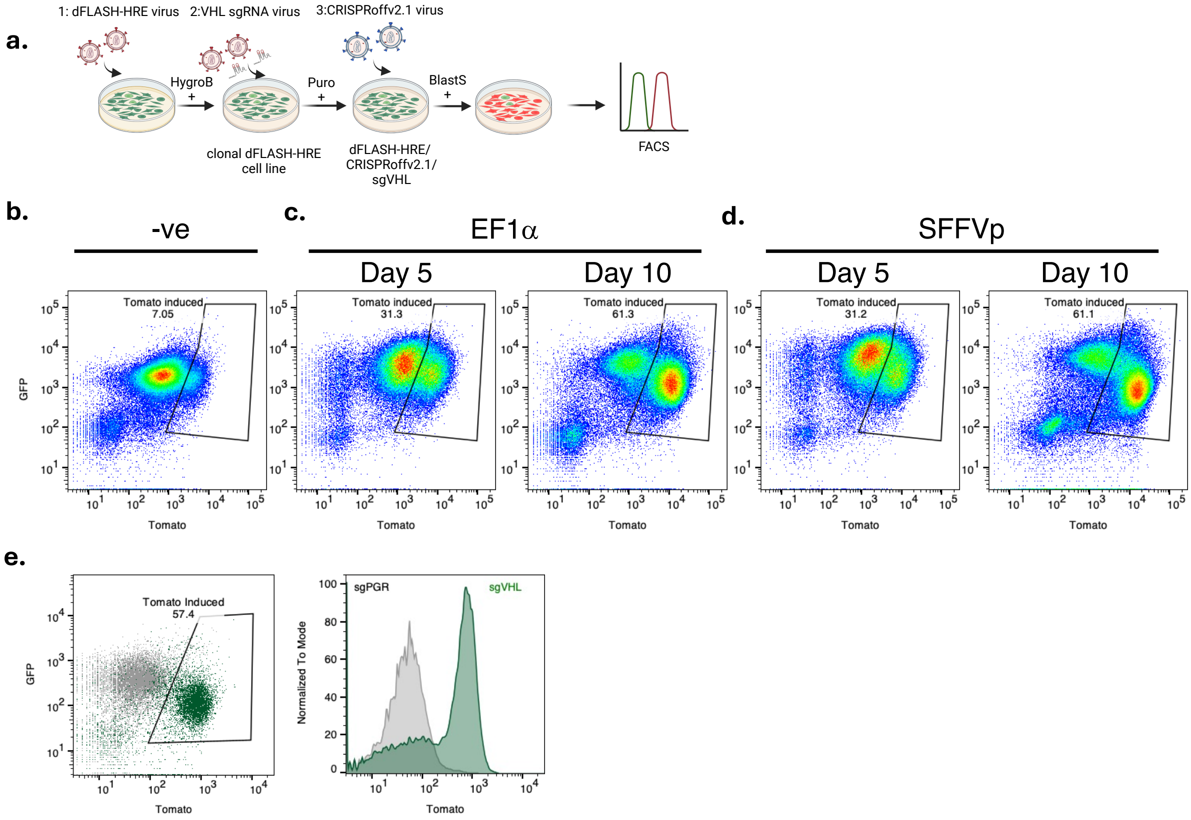

Supplementary Fig. 7. CRISPRoff mediated VHL knockdown induces mcdFLASH-HIF reporter expression.

(**a**) HEK293T cells were first transduced with dFLASH-HRE and a clonal reporting line was derived after hygromycin (HygroB) selection. Monoclonal HEK293T dFLASH-HRE cells were in turn transduced with the *VHL* sgRNA lentivirus (or the negative control guide *PGR* in (**e**)) and selected with puromycin (Puro). This line was then transduced with EF1α (left panels) or SFFVp (right panels) driven CRISPRoffv2.1 and selected with Blasticidin S (BlastS). Polyclonal populations were subjected to flow cytometry after 5 days or 10 days of selection for analysis of reporter expression. Created in BioRender. Peet, D. (2025) https://BioRender.com/c52b696 . (**b-d**) Dot plots for dFLASH expression from the (**b**) non-CRISPRoff parental line, (**c**) EF1α-CRISPRoffv2.1 transduced and (**d**) SFFVp-CRISPRoffv2.1 transduced populations after 5 or 10 days of blasticidin selection (see also Fig. 4**)**. (**e**) Replicate experiment of SFFVp driven CRISPRoffv2.1 as described in (**a-d**) with a sgPGR promoter targeting sgRNA as a control. Flow cytometry was performed 10 days after selection with Blasticidin S. Data are representative of n = 2 independent experiments.

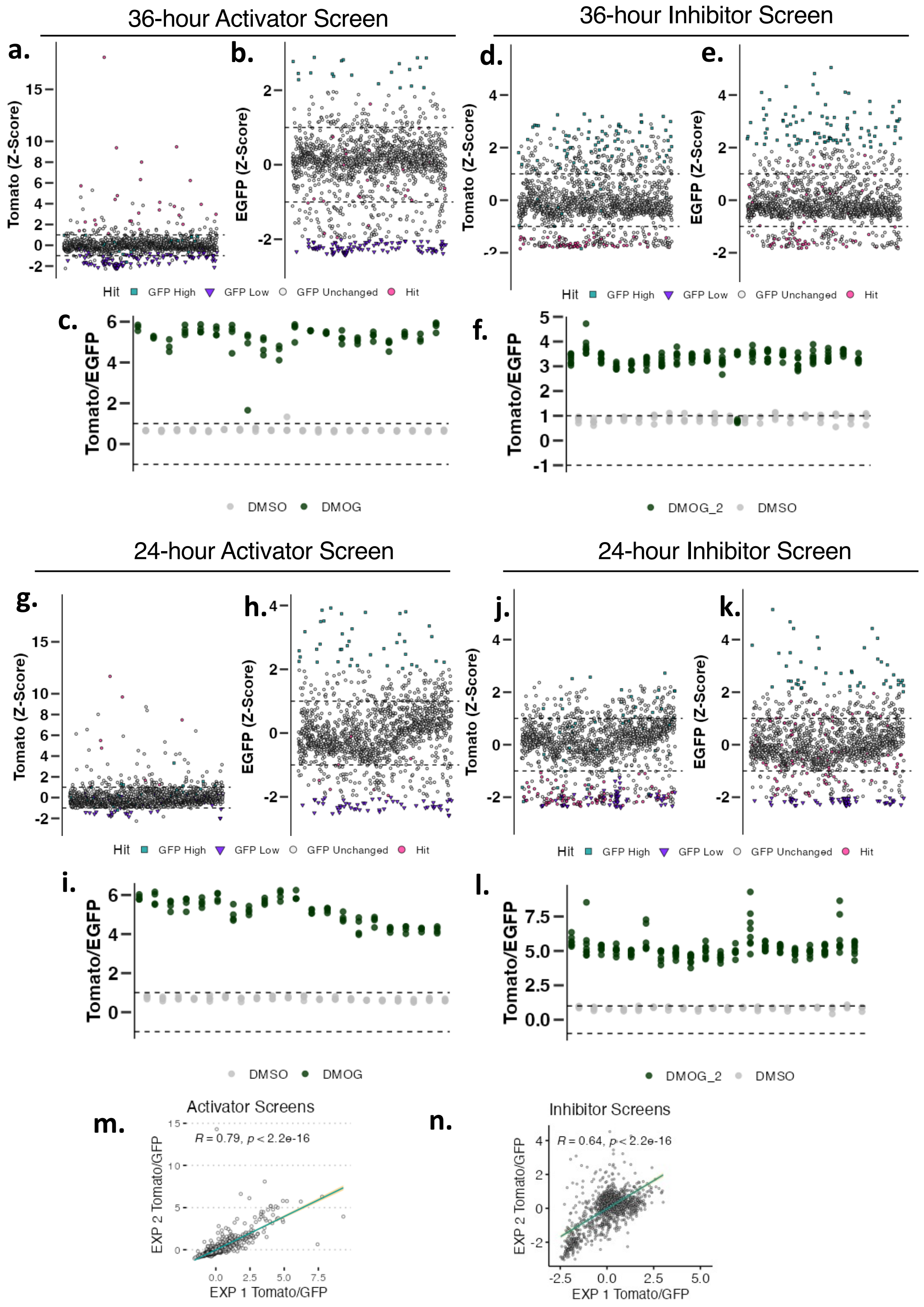

Supplementary Fig. 8. Hit selections and assessment of bimodal screen reproducibility between independent screens for activators and inhibitors of HIF-1α.
Compound-induced dFLASH-HIF reporter activity was used to score hits from the (**a-f**) 36-hour or the (**g-l**) 24-hour bimodal screens. Compound and control mean fluorescence intensity (MFI) from the (**a-c, g-h**) activator screen or (**d-f, j-l**) inhibitor screen. (**a,d, g, j**) Tomato MFI Z-score (**b, e, h, k**) GFP MFI Z-score of all compound treated wells and (**c, f, i , l**) Tomato/GFP MFI values from control treated wells. Lines indicate cut offs for hit criteria with hits shown in pink circles for each metric and dismissed compounds that change EGFP >±2SD shown in aqua squares (high GFP) or purple down-pointing triangle (low GFP), grey circles indicate unchanged Tomato/GFP (>±2SD). Dot plots in **(c, f, I, l)** indicate vehicle (grey) or DMOG (green) treated control wells, DMOG label refers to control wells in the activator screen, DMOG_2 refers to control wells in the subsequent inhibitor screen. (**m, n**) Pearson correlations of the Tomato/EGFP values between the 36-hour and the 24-hour screens for (**m**) reporter activation (R = 0.79, $p < 2.2\times10^{-16}$) or (**n**) reporter inhibition (R = 0.64, $p < 2.2\times10^{-16}$) for all 1595 compounds screened. Line of best fit indicated (green), yellow boundary is 95% confidence interval. Each compound screened in singlet across n = 2 independent screens. Source data are provided as a Source Data file.

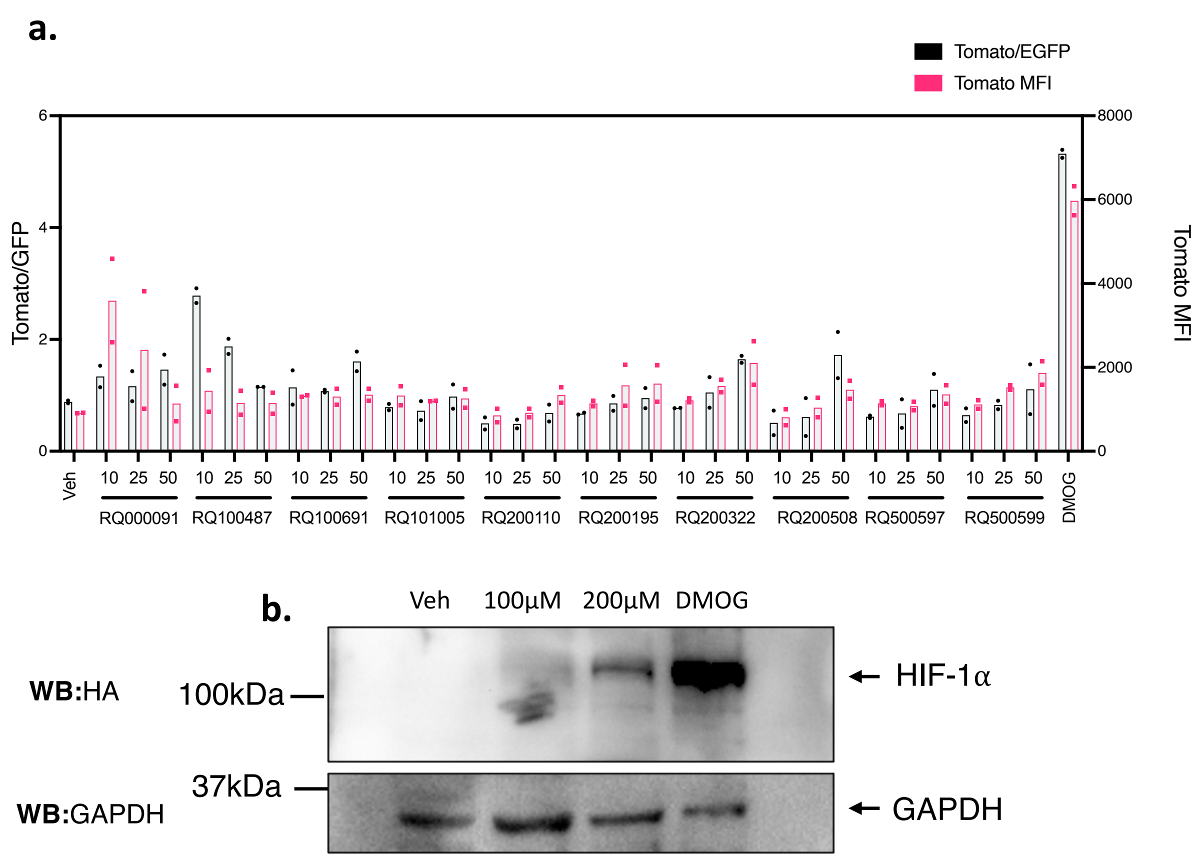

Supplementary Fig. 9. Rescreening of activator hits from 1595 compound small molecule screen reveals RQ200674 causes normoxic stabilisation of HIF-1α.

(**a**) The 11 top performing hits from the activator screens, excluding RQ200674 (presented in Fig. 7d) were rescreened against HEK293T mcdFLASH-HIF at 10 μM, 25 μM and 50 μM. Comparisons between Tomato/GFP and Tomato MFI dFLASH induction shown against vehicle (-ve ctrl) and 1 mM DMOG (+ve ctrl) treated populations (n= 2 independent biological experiments, each in duplicate. Presented as mean $\pm$ standard deviation).). (**b**) Immunoblot of whole cell extracts from HEK293T cells with endogenously HA-Flag tagged HIF-1α and treated as indicated with vehicle (0.1% DMSO),1 mM DMOG (+ve ctrl), or 100 μM and 200 μM of RQ200674 for 18 hours. Representative of n = 2 independent experiments. Source data are provided as a Source Data file.

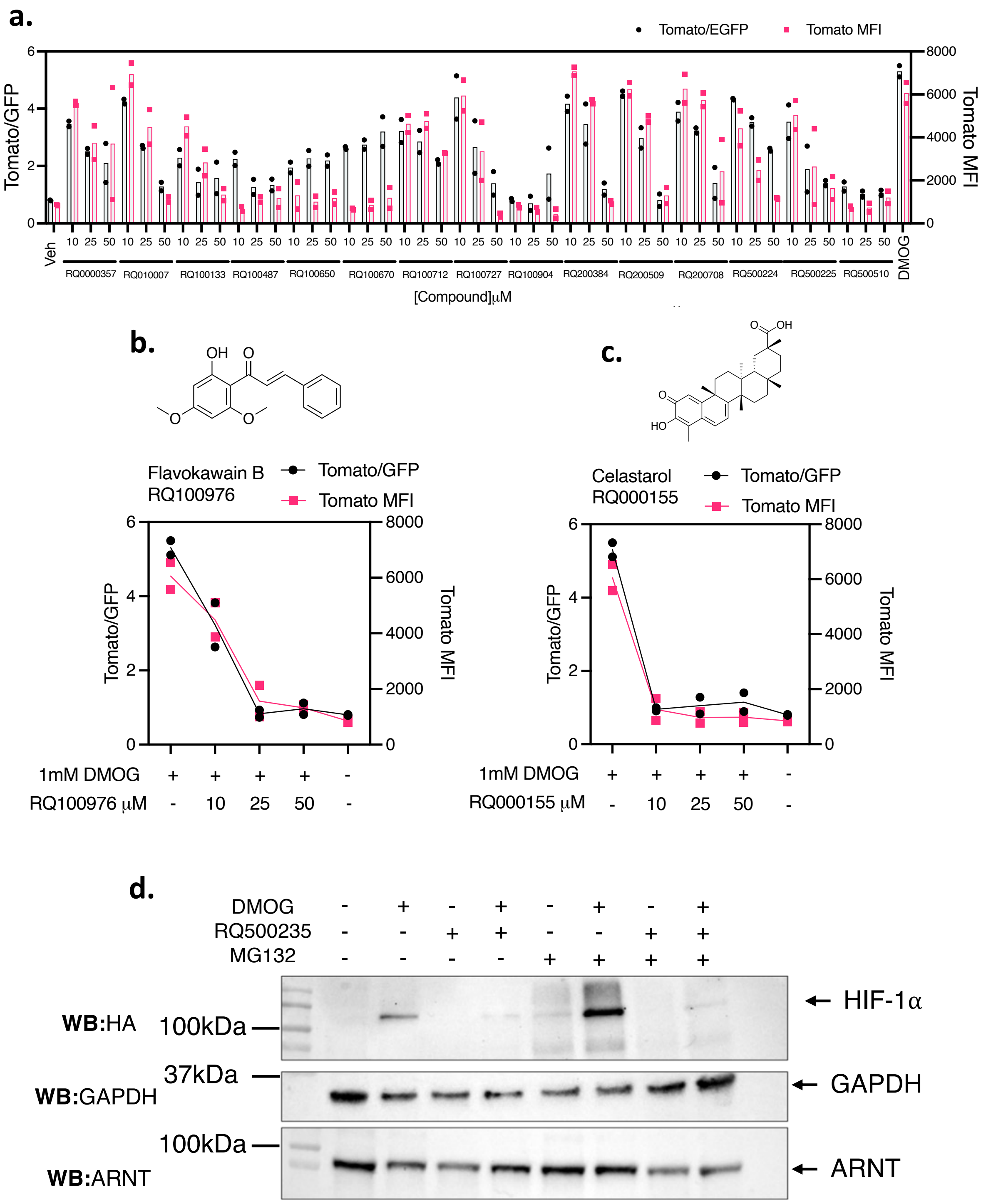

Supplementary Fig. 10. Flavokawain B, Celastarol and RQ500235 decrease dFLASH-HIF activity, while proteasomal inhibition does not rescue RQ500235-mediated decrease in HIF-1α protein levels.

(**a-c**) The top inhibitory compounds from inhibitor screening, including (**b**) Flavokawain B (RQ100976) and(**c**) Celastarol (RQ000155) were rescreened against dFLASH-HIF at 10 μM, 25 μM and 50 μM in 1 mM DMOG treated HEK293T dFLASH-HIF cells (24 hours), RQ500235 is presented in Fig. 7a. Comparisons between Tomato/GFP and Tomato MFI dFLASH induction shown against 0.1% DMSO (-ve ctrl) and 1 mM DMOG (+ve ctrl) treated populations (**a-c,** n = 2 biological replicates). (**d**) Immunoblot of whole cell extracts from HEK293T cells with endogenously HA-Flag tagged HIF-1α following a 12 hr treatment period with the indicated combinations of 1 mM DMOG (full 12 hr), 50 μM RQ500235 (final 6 hr) and 10 μM MG132 (final 3 hr). Representative of 2 independent experiments. Source data are provided as a Source Data file.

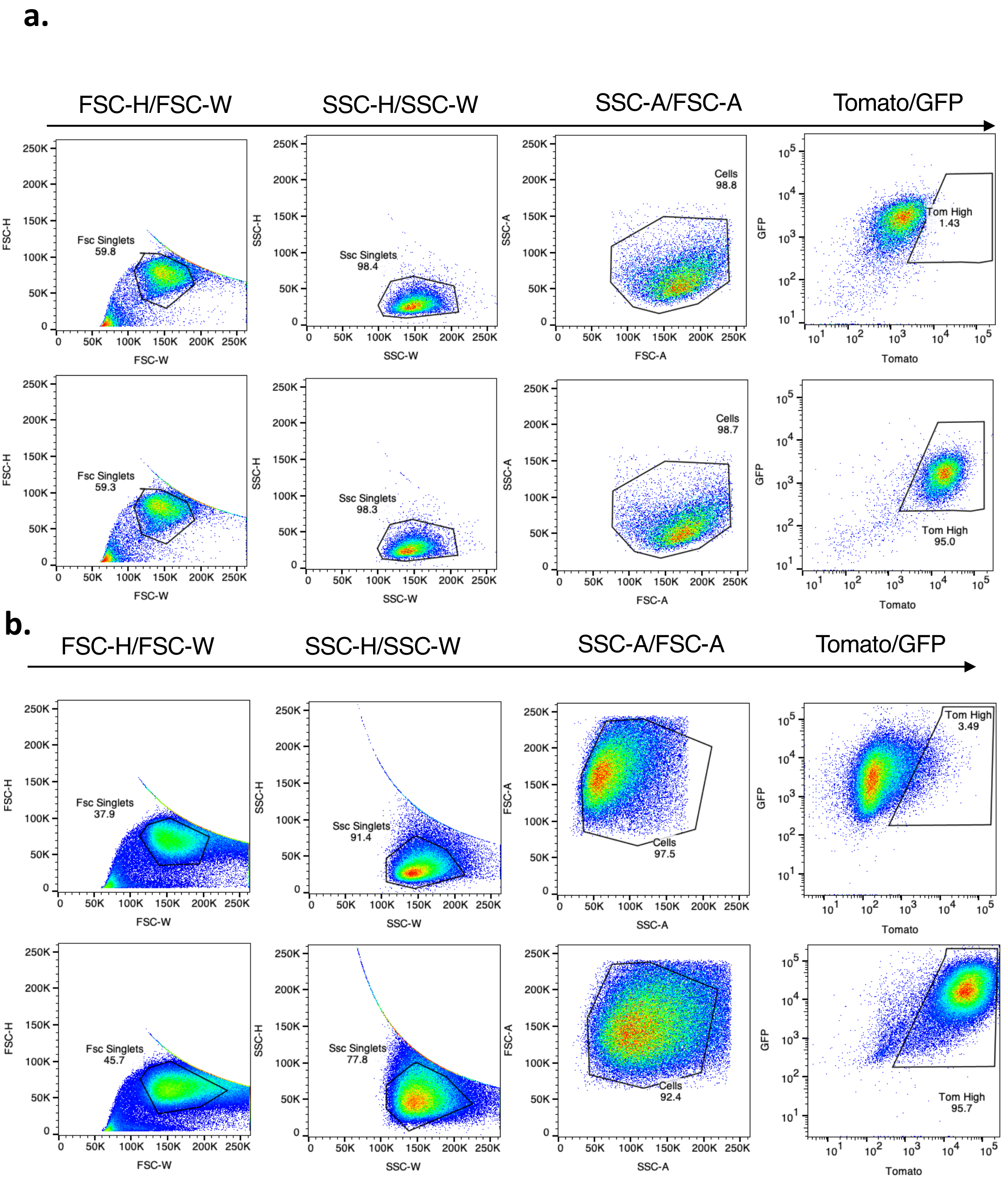

Supplementary Fig. 11 Representative plots for FACS gating strategies. Representative plots of the gating strategy (see Methods) for vehicle (0.1% DMSO, top row panels) and ligand treated (bottom row) monoclonal (**a**) HEK293T mcdFLASH-HRE cells and (**b**) T47D mcdFLASH-PRE cells. Populations are from Fig. 3.

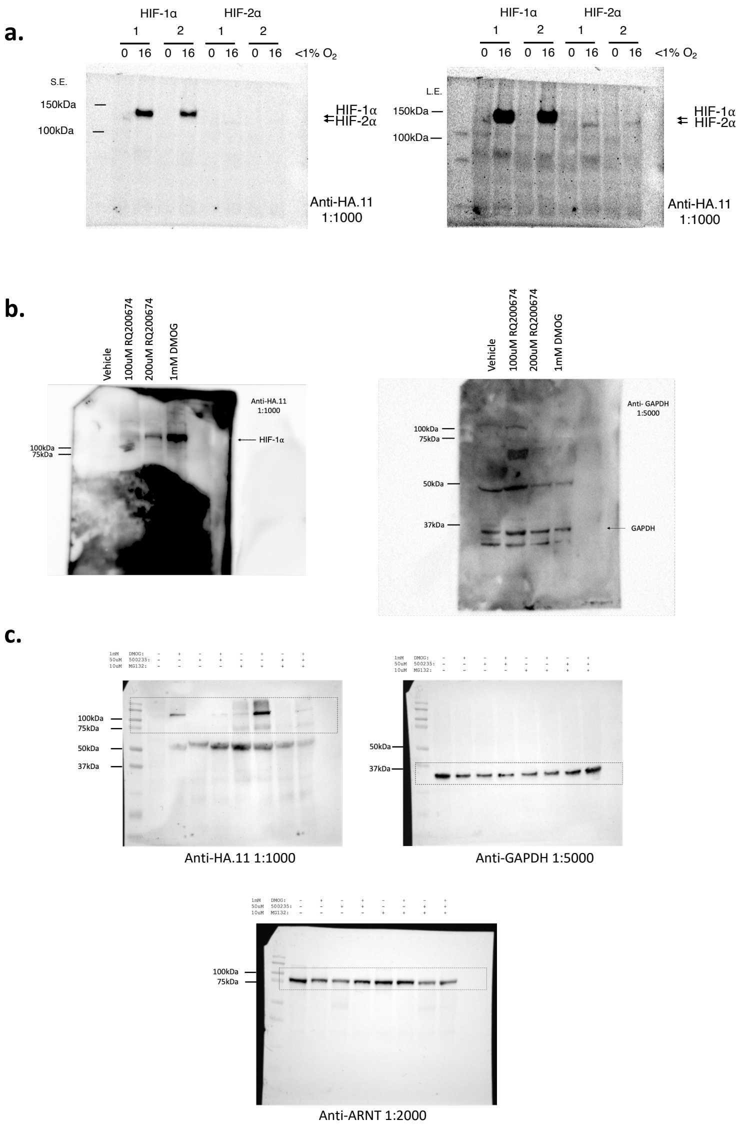

Supplementary Fig. 12. Representative Uncropped Western blots. (**a**) Supplementary Fig. 6a. (**b**) Supplementary Fig. 9b. (**d**) Supplementary Fig. 10d.

Supplementary Table 1: Synthetic toolkit for generation of reporter cell lines

Deposit Name:	Availability	Purpose
**Dual fluorescent reporter constructs:**		
pLV-REPORT(EF1α)	Addgene #172326	Reporter with mnucTomato and EF1α downstream promoter
pLV-REPORT(EF1α)-TTN	Addgene #172327	Reporter with mnucTomato-HSVtk-2A-NeoR and EF1α downstream promoter
pLV-REPORT(PGK)	Addgene #172328	Reporter with mnucTomato-HSVtk-2A-NeoR and PGK downstream promoter
pLV-REPORT(PGK/CMV)	Addgene #172330	Reporter with mnucTomato-HSVtk-2A-NeoR and PGK/CMV downstream promoter
12xHRE-pLV-Report-EF1α	Addgene: #172333	Reporter with HRE enhancer
12xHRE-pLV-REPORT(PGK)	Addgene #172334	Reporter with HRE enhancer
12xHRE-pLV-REPORT(PGK/CMV)	Addgene #172335	Reporter with HRE enhancer
PREcat-pLV-REPORT(PGK/CMV)	By Request	Reporter with a PR-responsive concatemer, with enhancers from 5 target genes, containing 6 PR response elements.
5xGRE-pLV-REPORT(PGK/CMV)	Addgene #172336	Reporter with GRE enhancer
12xHRE-pLV-REPORT(EF1α)	By Request	Reporter with HRE
12xHRE- pLV-REPORT(EF1α)-tdnucTomato	By Request	Reporter with tdnucTomato and EF1α downstream promoter
**Protein expression constructs:**		
pLV-TET2Puro	By Request	Doxycycline-inducible expression vector
pLV-TET2BlastR	By Request	Doxycycline-inducible expression vector
pLV-TET2nucTomato	By Request	Doxycycline-inducible expression vector
pLV-TET2Puro-gal4DBD-miniVPR-HA	Addgene #207171	Doxycycline-inducible expression vector for GAL4DBD-miniVPR
pLV-TET2Puro-gal4DBD-HIFCAD	Addgene #207173	Doxycycline-inducible expression vector for GAL4DBD-HIFCAD (727-826) with Myc tag
pEF-IRES-puro6 gal4DBD-HIFCAD myc tag	Addgene #207171	Constitutively expresses GAL4DBD-HIFCAD (727-826) with Myc tag
pEF-IRES-puro6 gal4DBD-HIFCAD pGalO linker	Addgene #207172	Constitutively expresses GAL4DBD-HIFCAD (727-826) with Myc tag
pENTR1a-CRISPRoffv2.1	Addgene #207174	Lentiviral expression vector for CRISPRoffv2.1 with BFP tag
pLV-EgI-NeoR	Addgene #207175	Gateway-compatible lentiviral expression plasmid with Neomycin resistance
pLV-EgI-BlasR	Addgene #207176	Gateway-compatible lentiviral expression plasmid with Blasticidin resistance
pLV-EgI-HygroR	Addgene #207177	Gateway-compatible lentiviral expression plasmid with Hygromycin resistance
pLV-EgI-ZeoR	Addgene #207178	Gateway-compatible lentiviral expression plasmid with Zeocin resistance
pLV-TET2BlastR-Cas9-TagBFP	Addgene #236726	Lentiviral delivered Inducible Cas9-TagBFP expression
pENTR1a-Cas9-TagBFP	Addgene #236725	Gateway-compatible Cas9-TagBFP cloning

Supplementary Table 2. dFLASH Bimodal high throughput screen details

Category	Parameter	Description
Assay	Type of assay	High Content, Live cell screen of a genetic reporter measuring nuclear TF-dependent Tomato expression and constitutive EGFP expression
	Target	HIF-1α-dependent Tomato expression
	Primary measurement	Ratio of Tomato expression to EGFP expression.
	Key reagents	HEK293T mcdFLASH-HIF reporter cells and 1mM DMOG in DMSO.
	Assay protocol	Assay protocol can be found in detail in the methods iunder "*High Content Imaging (HCI)"* and *"Bimodal small molecule screen to identify activators or inhibitors of the hypoxic response pathway.*"
	Additional comments	**Fig. 1** provides a schematic for the genetic reporter.
Library	Library size	1595 compounds supplied at 5mM in 1 μL of DMSO that were dried onto the plates.
	Library composition	Library was a mixture of synthetic and natural product compounds curated by Prof. Ronald Quinn.
	Source	Compounds were sourced from Compounds Australia (www.compoundsaustralia.com)
	Additional comments	
Screen	Format	96-well plates. Plates supplied were 20 96-well Costar CLS3603 black plates with clear bottoms.
	Concentration(s) tested	For Activation screening, 50μM for each compound was investigated. For Inhibition screening 25μM was investigated.
	Plate controls	At each timepoint, compound-free well with equivalent 0.1% DMSO (negative control) and 1 mM DMOG (positive control) were included.
	Reagent/ compound dispensing system	Compounds were dispensed into the 96 well format by Compounds Australia. Cells and DMSO or DMOG were added manually.
	Detection instrument and software	Thermofisher ArrayScan XTI was the imagining instrument. HCS Studio 3.0 was the primary analysis software for detection and quantification of nuclear fluorescence.
	Assay validation/QC	Z' > 0.5 for each screen were confirmed, as was ensuring hits met >2SD parameter for Tomato/GFP in **Supplementary Fig. 8**.
	Correction factors	N/A
	Normalization	Data was Z score normalized.
	Additional comments	
Post-HTS analysis	Hit criteria	Hit criteria is described in method section *"Bimodal small molecule screen to identify activators or inhibitors of the hypoxic response pathway"*. For activator screens, hits had to be >2SD for Tomato/EGFP and >1SD Tomato MFI Z score while EGFP expression did not change more than 2SD relative to mean of the compound treated population. For inhibitor hits criteria was <2SD for Tomato/EGFP and Tomato MFI, while again EGFP did not change more than 2SD relative to the compound treated population.
	Hit rate	36 hour activator screen: 25 compounds (1.4%)<br>24 hour activator screen: 8 compounds (0.5%)<br>Overall activator screen: 3 compound replicated between screens (0.18%)<br>36 hour inhibitor screen: 69 compounds (4.2%)<br>24 hour inhibitor screen: 81 compounds (5.07%)<br>Overall inhibitor screen: 36 compounds replicated between screens (2.25%).
	Additional assay(s)	Replicate dFLASH High content assays were done at 24 hours on a subset of hits for activator and inhibitor compounds to confirm their activity (**Supplementary Fig. 9, 10**).
	Confirmation of hit purity and structure	Compounds were reordered through Compounds Australia (www.compoundsaustralia.com) to confirm identity prior to re-assay and downstream investigations.
	Additional comments	
