# Supplementary material for: dFLASH; dual FLuorescent transcription factor activity sensor for histone integrated live-cell reporting and high-content screening
Source: Nat Commun. 2025 Apr 7;16:3298. doi: 10.1038/s41467-025-58488-w (PMC11977238; doi:10.1038/s41467-025-58488-w)
Supplement: Supplementary file 2 — Description of Additional Supplementary Information [file 41467_2025_58488_MOESM2_ESM.docx]

**Supplementary Data 1. Comparison of the dFLASH platform with other analogous screening platforms.** Key dFLASH characteristics and screening metrics were compared and benchmarked.

**Supplementary Data 2. DNA sequences used for enhancer cloning.**

**Supplementary Data 3. sgRNA oligo sequences used for cloning.**

**Supplementary Data 4. DNA Sequences of qPCR and genomic PCR primers used.**

**Supplementary Movie 1. Single cell temporal dynamics of HEK293T mcdFLASH-HIF cells**

HEK293T mcdFLASH-HIF cells were seeded at 1x10^5^ cells/dish in Poly-D-Lysine coated plates overnight prior to imaging with spinning disk confocal microscopy at 40x magnification. Cells were imaged every 15 min for 48 hours for Tomato (Magenta) and EGFP (Green) expression. Time stamps are given in top left. Data are a representative field of view of n=2 independent experiments.

**Supplementary Movie 2. Single cell temporal dynamics of T47D mcdFLASH-PGR cells**

T47D mcdFLASH-PGR cells were seeded at 5x10^5^ cells/dish in Poly-D-Lysine coated plates overnight prior to imaging with spinning disk confocal microscopy at 40x magnification. Cells were imaged every 15 min for 48 hours for Tomato (Magenta) and EGFP (Green) expression. Time stamps are given in top left. Data are a representative field of view of n=2 independent experiments.
