# Supplementary material for: dFLASH; dual FLuorescent transcription factor activity sensor for histone integrated live-cell reporting and high-content screening
Source: Nat Commun. 2025 Apr 7;16:3298. doi: 10.1038/s41467-025-58488-w (PMC11977238; doi:10.1038/s41467-025-58488-w)
Supplement: Supplementary file 9 — Reporting Summary [file 41467_2025_58488_MOESM9_ESM.pdf]

nature portfolio

Corresponding author(s): David C. Bersten

Last updated by author(s): 22/5/25

Reporting Summary

Nature Portfolio wishes to improve the reproducibility of the work that we publish. This form provides structure for consistency and transparency in reporting. For further information on Nature Portfolio policies, see our Editorial Policies and the Editorial Policy Checklist.

Statistics

For all statistical analyses, confirm that the following items are present in the figure legend, table legend, main text, or Methods section.

n/a	Confirmed	
[ ]	[x]	The exact sample size (*n*) for each experimental group/condition, given as a discrete number and unit of measurement
[ ]	[x]	A statement on whether measurements were taken from distinct samples or whether the same sample was measured repeatedly
[ ]	[x]	The statistical test(s) used AND whether they are one- or two-sided<br>*Only common tests should be described solely by name; describe more complex techniques in the Methods section.*
[x]	[ ]	A description of all covariates tested
[x]	[ ]	A description of any assumptions or corrections, such as tests of normality and adjustment for multiple comparisons
[ ]	[x]	A full description of the statistical parameters including central tendency (e.g. means) or other basic estimates (e.g. regression coefficient) AND variation (e.g. standard deviation) or associated estimates of uncertainty (e.g. confidence intervals)
[ ]	[x]	For null hypothesis testing, the test statistic (e.g. *F*, *t*, *r*) with confidence intervals, effect sizes, degrees of freedom and *P* value noted<br>*Give P values as exact values whenever suitable.*
[x]	[ ]	For Bayesian analysis, information on the choice of priors and Markov chain Monte Carlo settings
[x]	[ ]	For hierarchical and complex designs, identification of the appropriate level for tests and full reporting of outcomes
[ ]	[x]	Estimates of effect sizes (e.g. Cohen's *d*, Pearson's *r*), indicating how they were calculated

Our web collection on statistics for biologists contains articles on many of the points above.

Software and code

Policy information about availability of computer code

Data collection	Data from High Content Imaging experiments was generated using HCS Studio v3.0.0 and ArrayScan XTI ((Thermofisher Scientific)<br>FACS analysis was done utilising FlowJo (v. 10.9.1) from data from LSRFortess or FACS ARIA2 (BD Biosciences)<br>Western blots were visualised with Chemidoc (BioRad). RT-qPCR with StepOnePlus (Applied Biosystems). Iron Binding assays were performed using Glomax Discover Plate Reader (Promega). high throuput sequencing was performed at South Australian Genome Center using Illumina Nextseq 550 machine.
Data analysis	Statistical analysis was done in base R (R version 4.2.2), with the drc R package (Ritz et al 2016, reference 72), graphing with ggplot2 (R), ggsci (R), ggpubr (R), tidyverse (R). or using Graphpad Prism (V. 9.0.0). FACS analysis with FlowJo v. 10.9.1. Western blots by ImageLab (v. 6.0.1). qPCR with StepOne Software v2.3. HCS StudioTM 3.0 Cell Analysis Software (ThermoFisher) was used for image segmentation and quantification. No original code was generated for this study. DEseq2 and MAGeck were used to analyse CRISPR screening expts.

For manuscripts utilizing custom algorithms or software that are central to the research but not yet described in published literature, software must be made available to editors and reviewers. We strongly encourage code deposition in a community repository (e.g. GitHub). See the Nature Portfolio guidelines for submitting code & software for further information.

Data

Policy information about availability of data

All manuscripts must include a data availability statement. This statement should provide the following information, where applicable:

- Accession codes, unique identifiers, or web links for publicly available datasets
- A description of any restrictions on data availability
- For clinical datasets or third party data, please ensure that the statement adheres to our policy

Data Availability. Source data are provided with this paper. Additional data, including full construct sequences, are available from Addgene (https://www.addgene.org/David_Bersten/) or from corresponding authors upon request. CRISPR sequencing data is available at https://www.ncbi.nlm.nih.gov/geo/query/acc.cgi?acc=GSE290538

Research involving human participants, their data, or biological material

Policy information about studies with human participants or human data. See also policy information about sex, gender (identity/presentation), and sexual orientation and race, ethnicity and racism.

Reporting on sex and gender	n/a
Reporting on race, ethnicity, or other socially relevant groupings	n/a
Population characteristics	n/a
Recruitment	n/a
Ethics oversight	n/a

Note that full information on the approval of the study protocol must also be provided in the manuscript.

Field-specific reporting

Please select the one below that is the best fit for your research. If you are not sure, read the appropriate sections before making your selection.

- [x] Life sciences
- [ ] Behavioural & social sciences
- [ ] Ecological, evolutionary & environmental sciences

For a reference copy of the document with all sections, see nature.com/documents/nr-reporting-summary-flat.pdf

Life sciences study design

All studies must disclose on these points even when the disclosure is negative.

Sample size	sample size n is defined as either the number of independent experiments or the number of independent wells.
Data exclusions	no data were excluded from the analyses
Replication	All statistical comparisons are from biological replicates experiments performed on at least two independent occasions
Randomization	n/a no randomisation was performed
Blinding	n/a blinded analysis of samples was not performed

Reporting for specific materials, systems and methods

We require information from authors about some types of materials, experimental systems and methods used in many studies. Here, indicate whether each material, system or method listed is relevant to your study. If you are not sure if a list item applies to your research, read the appropriate section before selecting a response.

Materials & experimental systems

n/a	Involved in the study	
☐	☒	Antibodies
☐	☒	Eukaryotic cell lines
☒	☐	Palaeontology and archaeology
☐	☒	Animals and other organisms
☒	☐	Clinical data
☒	☐	Dual use research of concern
☒	☐	Plants

Methods

n/a	Involved in the study	
☒	☐	ChIP-seq
☐	☒	Flow cytometry
☒	☐	MRI-based neuroimaging

Antibodies

Antibodies used	anti-HIF1a (BD Biosciences #910959)<br>anti-HA (HA.11, Biolegend #16B12)<br>anti-Tubulin (Serotec #MCA78G)<br>anti-GAPDH (Sigma #G8796)<br>anti-ARNT (Proteintech #14105-1-AP).
Validation	All antibodies have been Validated for the described purpose (i.e. Western Blotting) by the manufacturers.

Eukaryotic cell lines

Policy information about cell lines and Sex and Gender in Research

Cell line source(s)	HEK293T (ATCC CRL-3216)<br>HEPG2 (ATCC HB-8065)<br>T47D (ATCC HTB-133)<br>BT474 (ATCC HTB-20)<br>U2OS (STR validated)
Authentication	Each cell line was not authenticated specifically, other than confirmation that they appeared to, and grew in accordance with, published data and supplier-provided datasheets. T47D and BT474 cells were confirmed as steroid responsive.
Mycoplasma contamination	Cells tested negative for mycoplasma contamination by PCR.
Commonly misidentified lines<br>(See ICLAC register)	The cell line HEK293T cells have been previously reported on the ICAC register as commonly misidentified cell line.<br>This line was commercially purchased from the indicated supplier who performed validation of cell line prior to purchase.

Animals and other research organisms

Policy information about studies involving animals; ARRIVE guidelines recommended for reporting animal research, and Sex and Gender in Research

Laboratory animals	21 day old CBA x C57BL/6 F1 (CBAF1)
Wild animals	n/a
Reporting on sex	female mice were used for granulosa cell isolation
Field-collected samples	n/a
Ethics oversight	Experiments were approved by The University of Adelaide Faculty of Health and Medical Sciences Animal Ethics Committee and were performed in accordance with the Australian Code of Practice for the Care and Use of Animals for Scientific Purposes; ethics number M-2023-074.

Note that full information on the approval of the study protocol must also be provided in the manuscript.

Plants

Seed stocks	n/a
Novel plant genotypes	n/a
Authentication	n/a

Flow Cytometry

Plots

Confirm that:

☒ The axis labels state the marker and fluorochrome used (e.g. CD4-FITC).

☒ The axis scales are clearly visible. Include numbers along axes only for bottom left plot of group (a 'group' is an analysis of identical markers).

☒ All plots are contour plots with outliers or pseudocolor plots.

☒ A numerical value for number of cells or percentage (with statistics) is provided.

Methodology

Sample preparation	Cells were trypsinised, washed in complete media (specified in methods) and resuspended in either;<br>1. flow cytometry sort buffer (Ca2+/Mg2+-free PBS, 2%FBS) prior to cell sorting or<br>2. flow cytometry analysis buffer (Ca2+/Mg2+ free PBS, 2%FBS, -/+ 1mM EDTA)
Instrument	BD Biosciences BD LSRFortessa<br>Biosciences FACS ARIA2
Software	Flowjo (v. 10.9.1)
Cell population abundance	Cell populations were identified from a minimum of 10,000 gated objects and estimated using untreated or untransduced cells using FlowJo software.
Gating strategy	Cell populations were gated by FSC-W/FSC-H, then SSC-W/SSC-H, followed by SSC-A/FSC-A prior to EGFP and Tomato detection. EGFP fluorescence was measured by a 530/30nm detector, and the Tomato fluorescence was determined with the 582/15nm detector. No compensation was required.

☒ Tick this box to confirm that a figure exemplifying the gating strategy is provided in the Supplementary Information.
